# From Stents to Smart Implants Employing Biomimetic Materials: The Impact of 4D Printing on Modern Healthcare

**DOI:** 10.3390/biomimetics10020125

**Published:** 2025-02-19

**Authors:** Antreas Kantaros, Florian Ion Tiberiu Petrescu, Theodore Ganetsos

**Affiliations:** 1 Department of Industrial Design and Production Engineering, University of West Attica, 12244 Athens, Greece; ganetsos@uniwa.gr; 2 “Theory of Mechanisms and Robots” Department, Faculty of Industrial Engineering and Robotics, National University of Science and Technology Polytechnic Bucharest, 060042 Bucharest, Romania

**Keywords:** 4D printing, healthcare, bioprinting, self-healing implants, smart stents, personalized drug delivery, adaptive prosthetics, medical devices, material innovation, artificial intelligence

## Abstract

The sector of 4D printing represents a new frontier in additive manufacturing that allows for a material’s capability to adapt and respond to various stimuli, such as thermal transitions, humidity, and pH levels. The adaptability of such a material has great potential in healthcare applications, especially in designing personalized and responsive medical devices. This article looks into the revolutionary potential of healthcare applications of 4D printing, referencing applications in self-repairable implants, smart stents, personalized drug delivery systems, and response-based prosthetic devices. The advances in 3D printing have created a platform for such innovations to take place, while the material properties unique to 4D printing allow new methods of tackling existing health issues. However, the large-scale application of 4D printing in medicine is currently hampered by material limitations, regulation challenges, and financial challenges. In spite of these challenges, ongoing advances in technologies, combined with artificial intelligence and machine learning, provide the potential to surpass such challenges, hence improving the precision, efficacy, and personalization of medical devices. This work outlines existing applications, looks at potential areas of application in the future, and analyzes potential applications of 4D printing contributing to healthcare, recognizing challenges that need to be overcome in order to unlock its full potential.

## 1. Introduction

The ongoing advancement of printing technology has initiated a novel phase of innovation in healthcare, with 3D printing remaining at the forefront of fabricating highly tailored medical devices and implants. Nonetheless, with technological advancements, the emersion of 4D printing is transforming the potentialities in medical design [1]. Four-dimensional printing extends the principles of 3D printing by incorporating an additional dimension—time [2,3,4]. This dimension enables printed materials to alter their shape and function in reaction to environmental stimuli, such as temperature, moisture, light, or pH levels. These adaptable materials, which may react to environmental conditions, possess significant potential applications in healthcare, especially in the development of self-healing implants, bio-implants, and responsive medical devices [5]. The influence of 4D printing is apparent in the development of advanced and functional medical items, as well as in the capacity to create devices that can adjust to the dynamic requirements of the human body. This work examines the applications of 4D printing in healthcare, emphasizing biomimetic materials utilized in stents, implants, and customized medical devices, and investigates how this technology can transform contemporary healthcare procedures [6].

Unlike traditional 3D printing, where objects are fabricated layer by layer using static materials, 4D printing leverages the concept of materials that can respond and change according to specific external stimuli. These stimuli can be as varied as temperature fluctuations, humidity levels, light exposure, or even pH changes, making 4D printing incredibly versatile for applications where adaptability and flexibility are crucial [7]. The materials used in 4D printing are often smart materials or stimuli-responsive materials, which are specifically designed to react to environmental changes by altering their shape, structure, or properties. This is achieved through the use of materials like hydrogels, shape-memory polymers (SMPs), and biocompatible composites, which have the ability to return to their original state after being deformed, or change properties in response to external conditions [8].

The applications of 4D printing are vast and particularly significant in fields like healthcare, where devices or implants can be designed to adapt to dynamic bodily conditions [9]. For example, a 4D-printed stent can expand or contract in response to temperature changes, such as those induced by body heat, allowing for more precise and effective treatments [10,11,12]. In the case of smart implants or bio-implants, 4D printing can create materials that adjust to the body’s healing process, ensuring a more natural integration with tissues [13]. Additionally, this technology can be used to create drug delivery systems that release medications in response to changes in the body’s environment, such as pH or temperature, offering a level of personalization that was previously unattainable [14]. Thus, 4D printing introduces a novel approach to medical devices, bringing them closer to the ideal of being responsive, adaptive, and personalized, all of which contribute to improved patient outcomes [15,16].

Current trends in healthcare point toward a growing interest in personalized and precision medicine, and 4D printing is poised to play a central role in this movement. As medical technology continues to evolve, there is an increasing emphasis on patient-specific devices that not only match the anatomical requirements of individuals but also adapt to changing conditions over time [17]. As stated earlier, 4D printing makes this possible by utilizing materials that can undergo controlled shape changes, ensuring that implants and devices are not static but can adjust to environmental stimuli, including body temperature, pH, or even mechanical forces. One of the most recent evolvements is the potential for self-healing implants, which can repair themselves in response to damage or wear, a crucial feature for devices used in long-term applications [18]. Additionally, the integration of biomimetic materials—materials inspired by biological processes or natural systems—into 4D printing is transforming the design of medical devices, providing more natural interfaces between implants and the human body. By mimicking the responsive properties of biological tissues, these biomimetic materials improve the compatibility and longevity of implants, making them more efficient in promoting healing [19]. The ability to design adaptive medical solutions that respond in real time to the needs of the body is driving the development of next-generation healthcare technologies, with potential applications ranging from neurosurgery to cardiovascular health, and extending into drug delivery systems, tissue engineering, and beyond.

This study primarily aims to investigate the revolutionary potential of 4D printing in medical devices and bioprinting, focusing on how these sophisticated technologies are redefining healthcare practices. Unlike prior works that mainly address the principles of 4D printing or its broad applications, this study offers a concentrated and thorough examination of its significance in personalized and adaptive medical treatments. It specifically underscores the incorporation of biomimetic materials, self-healing implants, and intelligent medication delivery systems, stressing how 4D printing transcends conventional 3D printing in the fabrication of dynamic, patient-specific devices. This review critically assesses recent achievements, emerging trends, and significant scientific and technological discoveries, focusing on the relationship between material innovation and practical therapeutic applications. Additionally, it analyzes the legal and translational obstacles that hinder the extensive implementation of these technologies, offering a prospective view on their future significance in precision medicine. Through this comprehensive analysis, this review not only synthesizes existing knowledge but also presents novel insights into how 4D printing is redefining the landscape of modern healthcare.

## 2. The Evolution of Bioprinting and Medical Devices

The rapid progression of 3D printing technology over the last few decades has significantly shaped the landscape of healthcare, laying the foundation for the more advanced 4D printing applications we see today [20]. Early advancements in 3D printing focused primarily on materials like plastics, metals, and ceramics, which were used to fabricate prosthetics, orthopedic implants, and even surgical guides [21,22]. The ability to rapidly prototype and create customized implants allowed for greater precision and fit, improving the overall efficacy of medical devices. In addition, 3D printing reduced the time and cost associated with traditional manufacturing methods, allowing for more efficient production processes [23]. As the technology matured, its applications expanded into more complex fields, such as bioprinting, where living cells are used to print tissues and organ-like structures, pushing the boundaries of what could be achieved with conventional methods. These developments paved the way for 4D printing by demonstrating that not only could medical devices and implants be tailored to individual patients, but they could also be printed using materials that could respond to stimuli like temperature, moisture, or pH [24].

While 3D printing provided the initial technological breakthrough in healthcare, the evolution toward 4D printing was driven by the demand for even more sophisticated and dynamic solutions. One of the key limitations of traditional 3D-printed medical devices was their static nature—once printed, the components were fixed in shape and functionality. However, as researchers began exploring the integration of responsive materials into 3D printing processes, the concept of 4D printing emerged [25]. This has been particularly useful in the development of bio-implants, stents, and drug delivery systems that can adjust dynamically to the body’s changing conditions, such as temperature, pH, or stress levels. Through this progression, smart materials—such as hydrogels, shape-memory alloys, and stimuli-responsive polymers—are now being incorporated into the design and fabrication of medical devices, enabling them to perform tasks that were previously unattainable [25].

One of the most significant impacts of 4D printing in the medical field is its ability to create adaptive medical devices that are not only tailored to the specific needs of a patient but also capable of adjusting their behavior over time to optimize performance. For instance, drug delivery systems can release medication at precise times or locations within the body based on local changes in pH or other stimuli [26]. This evolution from static devices to those with the ability to adapt has significant potential for improving patient outcomes, reducing the need for follow-up surgeries and enhancing the efficiency of treatments [27]. Furthermore, the introduction of self-healing materials in 4D printing has made it possible to develop implants and prosthetics that can repair themselves in response to damage or wear, reducing the likelihood of implant failure and minimizing the need for surgical intervention [28]. The ability of 4D printing to create such intelligent, responsive systems has revolutionized the design and function of medical devices by enabling more personalized, efficient, and long-lasting healthcare solutions. For example, the creation of bio-implants using 4D printing allows for materials that interact with biological tissues, changing shape and functionality to better integrate with the body and enhance healing processes [29,30]. Moreover, biosensors integrated into 4D-printed implants can monitor various environmental factors, sending real-time data to healthcare providers to optimize treatment plans [31]. In this way, the convergence of additive manufacturing and smart materials has significantly pushed the boundaries of what medical devices can achieve, offering new approaches to treat complex conditions such as tissue regeneration, vascular diseases, and orthopedic injuries. As the technology continues to evolve, it is anticipated that 4D printing will not only further personalize medical treatments but also contribute to the development of next-generation healthcare solutions that provide more efficient, effective, and patient-centric care. Table 1 presents a relevant comparison of 3D and 4D printing in medical device evolution.

## 3. Key Applications of 4D Printing in Healthcare

As mentioned before, the integration of 4D printing into healthcare is revolutionizing the design and functionality of medical devices by enabling materials to not only retain a fixed shape but to transform and adapt in response to specific biological stimuli over time. This technology opens up new possibilities for medical applications, where adaptability, self-repair, and real-time response to the environment can significantly enhance patient care. Self-healing implants, smart stents, personalized drug delivery systems, and adaptive prosthetics are among the most promising applications of 4D printing in healthcare. These technologies leverage smart materials that respond to changes in temperature, moisture, pH, or mechanical stress, facilitating the creation of patient-specific solutions that can improve the efficiency and longevity of medical devices. This chapter will explore the most notable applications of 4D printing in healthcare, discussing the potential benefits of each technology, current developments, and their role in transforming medical practice and improving patient outcomes. Through these innovations, 4D printing holds the potential to change the future of healthcare treatments, offering solutions that are more adaptive, efficient, and personalized than ever before.

### 3.1. Self-Healing Implants

One of the most promising applications of 4D printing in healthcare is the development of self-healing implants, where 4D-printed materials possess the ability to repair themselves in response to stimuli, such as temperature, mechanical stress, or chemical signals. The concept of self-healing materials in medical devices offers substantial advantages, particularly in the context of bone scaffolds, wound healing, and implantable devices. Self-healing materials can effectively reduce the risks associated with mechanical degradation or damage that can occur over time in implanted medical devices. For example, in bone tissue engineering, 4D-printed bone scaffolds made from biodegradable polymers or composite materials can not only support the initial healing process but also autonomously repair small cracks or fractures that might otherwise compromise the scaffold’s structural integrity [32,33]. This capability significantly enhances the longevity and reliability of implants, particularly in environments where the body constantly exerts mechanical forces on the implant site, such as in bone fractures or joint replacements.

**Figure 1:**
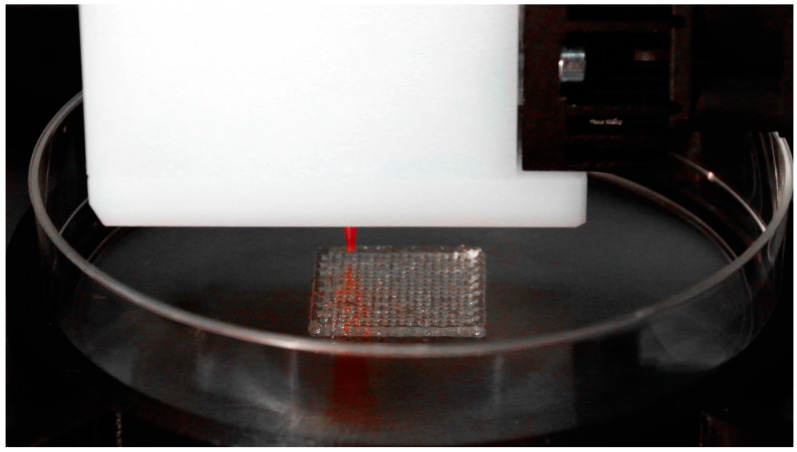
Tissue engineering scaffold fabricated with a 3D-Bioplotter [36].

The benefits of self-healing 4D-printed implants are multifold, ranging from improving the efficacy of the healing process to reducing the need for additional surgical interventions. By embedding self-repairing mechanisms within the implant’s structure, these implants can mitigate the complications that arise from wear and tear, infection, or poor integration with the host tissue [34]. In the case of wound healing, 4D-printed materials can be designed to release bioactive agents, such as growth factors or antimicrobial compounds, in response to environmental changes, facilitating the repair of tissue at the cellular level. In addition, 4D-printed wound dressings or bandages can adapt their properties (e.g., stiffness or porosity) based on the stage of wound healing, promoting faster recovery. This adaptability can further contribute to the patient’s comfort and reduce healthcare costs, as the need for constant monitoring and follow-up treatments may decrease [35]. The ability of these materials to self-repair not only enhances their practical application in medicine but also holds promise for reducing the long-term complications associated with medical implants and improving overall patient outcomes. Figure 1 depicts such an implant, while being fabricated by a bioplotter.

### 3.2. Smart Stents

The emergence of 4D printing has opened up new possibilities for the development of smart stents, medical devices that can dynamically respond to changes in their environment, such as body temperature, pH levels, or mechanical stresses. Traditional stents, used to support blood vessels or other ducts within the body, are typically made from metal alloys or polymers that maintain a fixed shape once implanted [37]. However, with the integration of 4D printing technologies, stents can now be fabricated with smart materials that adapt to environmental stimuli, offering enhanced functionality and improved outcomes for patients. For example, temperature-responsive materials can be engineered to expand or contract in response to the heat of the body, providing better integration with the surrounding tissue and ensuring more effective long-term performance. This ability to dynamically change shape or size in response to environmental changes not only improves the mechanical properties of the stent but also reduces the risk of complications, such as inflammation or restenosis (the renarrowing of a blood vessel) [38].

**Figure 2:**
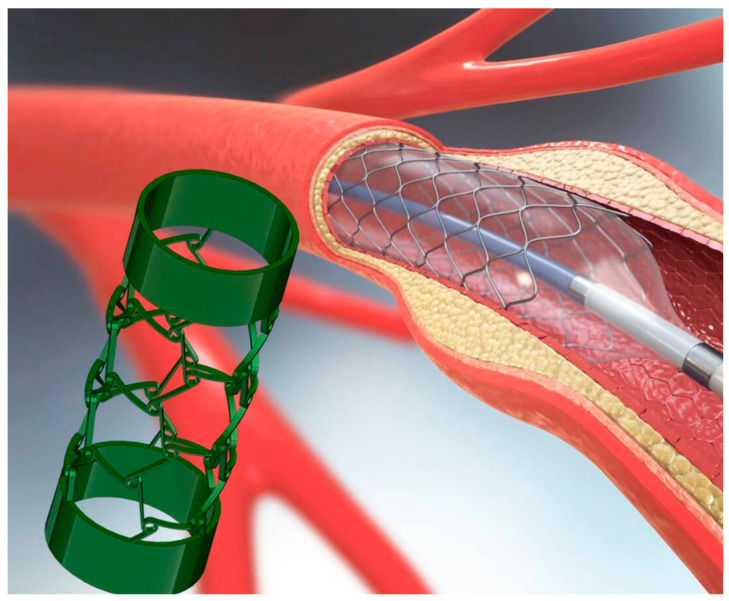
Illustration of stent implantation for supporting blood circulation into blood vessels [41].

The development of 4D-printed smart stents is particularly promising in the context of vascular stents, where adaptability can significantly improve their therapeutic effectiveness. By utilizing shape-memory alloys or hydrogel-based composites that are responsive to temperature, these stents can be designed to conform more precisely to the dimensions of the vessel once implanted [39]. This adaptability degree enables better patient-specific customization in stent placement, as well as the ability to optimize the mechanical force applied to the vessel wall, thus reducing the risk of further injury or excessive tissue damage. Moreover, smart stents that are able to adapt to other biological factors, such as changes in pH levels due to localized inflammation or tissue healing, could also help in minimizing the need for additional procedures or intervention. As a result, 4D-printed smart stents not only promise to enhance patient outcomes but could also lead to more personalized medicine, where stents are tailored to an individual’s specific biological and environmental conditions [40]. This approach is expected to significantly reduce the rates of stent failure and improve overall long-term treatment efficacy, particularly for cardiovascular diseases and other conditions requiring vascular support. Figure 2 depicts an illustration of such a 4D-printed stent implantation for supporting blood circulation into blood vessels.

### 3.3. Personalized Drug Delivery Systems

One of the most promising applications of 4D printing in healthcare lies in the development of personalized drug delivery systems that can release medication in response to specific internal body conditions. Traditional drug delivery systems often rely on a fixed release schedule, where drugs are administered at predetermined intervals, regardless of the patient’s physiological state [42]. However, 4D-printed systems offer the potential for more dynamic and responsive drug delivery, wherein materials are engineered to release therapeutic agents based on environmental stimuli such as pH, temperature, or biochemical signals [43]. By integrating these stimuli-responsive materials into drug delivery devices, 4D printing allows for the creation of systems that can tailor the release of drugs according to real-time changes in the body, ensuring more effective and targeted treatments. For instance, a pH-responsive drug delivery system could release drugs specifically in the acidic environment of the stomach or in response to the local conditions in areas of inflammation, offering a level of precision and control that traditional systems cannot provide.

Moreover, 4D-printed personalized drug delivery systems offer the ability to develop patient-specific therapies, which is especially beneficial for individuals with chronic conditions or those undergoing complex treatments. These systems can be designed to adapt their shape or structure in response to factors such as body temperature or metabolic changes, ensuring that drugs are delivered at the right time and in the correct dosage. For example, 4D-printed implants or microdevices could be used to release insulin or chemotherapeutic agents at precisely the right moment based on real-time glucose levels or tumor markers, providing personalized care and reducing the side effects associated with conventional treatments [44]. Furthermore, these advanced drug delivery systems can be integrated with biomimetic materials that respond to multiple stimuli simultaneously, offering even greater control over drug release [45]. This adaptive approach enables a level of flexibility and precision that has the potential to revolutionize treatments for conditions such as cancer, diabetes, and autoimmune diseases, paving the way for a new era of personalized medicine. By offering on-demand drug delivery that reacts to the individual needs of the patient, 4D printing ensures more effective treatments with fewer complications and an improved quality of life for patients.

### 3.4. Adaptive Prosthetics and Implants

In this context, 4D printing also exhibits high potential in the field of adaptive prosthetics and implants, enabling the creation of devices that not only function to restore lost body parts but also improve and evolve over time. Traditional prosthetics and implants are often designed to be static, requiring manual adjustments or replacements as a patient’s condition changes [46]. However, with 4D printing, prosthetic and implant designs can be made from stimuli-responsive materials that adapt and self-modify in response to changes in the body’s internal environment, such as temperature, pressure, or chemical signals [47]. This adaptability means that implants and prosthetics can respond to healing processes or wear and tear, gradually adjusting to enhance functionality and comfort. For example, an implant designed to support bone regeneration could adjust its stiffness as the bone heals, allowing for better integration with the surrounding tissue and reducing the risk of complications such as implant failure or rejection. Similarly, prosthetics can adapt to the user’s specific activities, providing more comfort and usability throughout daily tasks, potentially reducing the need for frequent adjustments or replacements.

The ability to design adaptive prosthetics and implants with 4D printing also holds the promise of enhancing patient outcomes by offering personalized, patient-specific solutions. These devices can be created with materials that respond to biomechanical feedback, such as the forces or movements exerted by the wearer, and optimize the fit and functionality over time. For example, an amputation site may change over time due to tissue growth or changes in muscle tone, and 4D-printed prosthetics can adjust accordingly, improving comfort and reducing the risk of pressure sores or irritation [48]. Additionally, adaptive implants that change their shape in response to environmental cues could be used in areas like orthopedic implants, where the device might progressively change its structure to provide better support as the surrounding bone strengthens or heals. Furthermore, 4D printing opens new possibilities in the creation of bioactive implants, which can release therapeutic agents, such as growth factors or anti-inflammatory drugs, in response to changes in the body, thus enhancing the healing process or preventing complications [49]. Ultimately, 4D-printed prosthetics and implants provide an innovative, personalized approach to healthcare, significantly improving long-term outcomes and ensuring better quality of life for patients. Table 2 provides a clear comparison of the key applications of 4D printing in healthcare, including their benefits and current research examples.

## 4. Challenges and Limitations

While the potential of 4D printing in healthcare is vast, its widespread implementation faces significant challenges. These challenges, spanning from material development, regulatory frameworks, and cost-effectiveness, present hurdles that must be overcome to ensure the success and sustainability of 4D-printed medical devices. Despite the promising applications in areas such as self-healing implants, smart stents, and personalized drug delivery systems, addressing these obstacles is crucial to bringing 4D printing from experimental research to everyday clinical use. In this chapter, we will explore the primary limitations currently faced by 4D printing technology in healthcare and the critical steps needed to overcome them. These include the need for advanced biocompatible and responsive materials, navigating complex regulatory approval processes, and the challenge of making 4D printing both economically viable and scalable for large-scale healthcare applications.

### 4.1. Material Constraints

One of the primary challenges facing the widespread adoption of 4D printing in healthcare is the limitation of materials that can respond effectively to external stimuli such as temperature, moisture, and pH. While 3D printing has already demonstrated the ability to produce complex structures, 4D printing introduces an additional layer of complexity by requiring materials that not only form the desired shapes but can also change their properties over time. This necessitates the development of “smart” materials, which can adapt and transform in response to specific environmental cues. In the context of healthcare, these materials need to be biocompatible, non-toxic, and capable of performing consistently within the human body [50]. The current materials used in 4D printing, such as certain polymers, hydrogels, and composites, show promise in laboratory settings, but they often fall short in terms of their long-term stability, responsiveness, and the ability to integrate with biological tissues. For example, while hydrogels can undergo swelling in response to water absorption, their mechanical strength and degradation rates can vary, making them less reliable for certain medical applications like implants or scaffolds for tissue engineering [51].

In addition to the need for responsiveness, 4D printing materials used in medical devices must adhere to strict biocompatibility standards [52]. The ideal materials would not only change shape or function in response to stimuli but also work harmoniously with the body’s biological systems without causing adverse reactions such as inflammation, immune response, or toxicity [53]. Currently, there is a gap in the availability of materials that meet these stringent requirements while also maintaining the mechanical properties necessary for medical devices. For instance, while some biomaterials such as shape-memory alloys or thermosensitive polymers show potential, they are often limited by factors such as mechanical performance, degradation rates, or the difficulty in achieving the desired responsiveness over a prolonged period [54,55]. As 4D printing technology advances, there is a critical need for interdisciplinary collaboration between material scientists, biomedical engineers, and clinicians to design new materials that are not only responsive to stimuli but also possess the strength, durability, and biocompatibility required for effective medical use. This material innovation is pivotal for realizing the full potential of 4D printing in applications like self-healing implants, adaptive prosthetics, and drug delivery systems.

### 4.2. Regulatory Hurdles

Regulatory hurdles present significant challenges to the widespread implementation of 4D-printed medical devices, as these technologies are still emerging and lack established frameworks for approval. Unlike traditional manufacturing methods, 4D printing involves materials and devices that change over time in response to environmental stimuli, complicating the regulatory process. The dynamic nature of 4D-printed medical devices introduces complexities in determining their safety, performance, and long-term effects. Current regulatory systems, such as those established by the FDA in the United States or the European Medicines Agency (EMA) in Europe, are primarily designed for static devices and materials that do not exhibit such adaptive behaviors [56]. As a result, regulatory agencies must develop new standards to address the unique challenges of 4D printing, including ensuring that these devices function as intended throughout their lifespan and do not cause unexpected harm to patients. This requires the creation of testing protocols that account for the materials’ transformation properties, as well as their interaction with biological systems under varying physiological conditions [56].

Moreover, the approval process for 4D-printed medical devices is hindered by the fact that regulatory agencies are often hesitant to grant approval for products with limited long-term data, especially those involving novel materials [57]. The incorporation of responsive materials in medical devices means that their behavior may vary depending on the environmental conditions within the body, introducing uncertainty into the evaluation process. For example, devices such as stents or implants that expand or contract based on body temperature or pH levels may not behave consistently over time, posing risks in clinical applications. Additionally, as 4D printing is often used in personalized medicine, the regulatory framework must also address the customization of devices for individual patients, which complicates the standardization required for mass approval. Consequently, the development of clear, adaptive regulatory pathways for 4D-printed medical devices is essential to ensure their safety, efficacy, and timely entry into the healthcare market. This may involve close collaboration between regulatory bodies, researchers, and manufacturers to establish appropriate testing methods, safety guidelines, and post-market surveillance mechanisms to monitor the long-term performance of 4D-printed devices [58].

Moreover, it is crucial to assess the ethical aspects of 4D printing in healthcare, more precisely personalized medicine. The ability to design and fabricate dynamic and personalized medical devices raises serious questions regarding patient consent, confidentiality of patients’ information, and fair access to new treatment alternatives. Proper informed consent of patients regarding the long-term effects of implants manufactured using 4D printing, such as their dynamic properties and potential hazards, is crucial in maintaining high ethical standards in healthcare provision. In addition, gaps in the provision of 4D-printed healthcare services would aggravate health disparities, hence making it even more crucial to put in place policies that promote affordability and accessibility. Addressing such issues is crucial in ensuring that 4D printing is used ethically to meet the needs of all patients regardless of their socioeconomic status.

### 4.3. Cost and Scalability

The adoption of 4D printing technology in healthcare is significantly hindered by challenges related to cost-effectiveness and scalability. While 4D printing holds immense potential for revolutionizing medical devices and personalized healthcare solutions, the production costs associated with this technology remain high. One of the primary reasons for the high cost is the advanced materials required for 4D printing, which must possess specific properties such as biocompatibility, responsiveness to stimuli, and durability. These materials, often custom-designed for specific applications, are more expensive than traditional biocompatible materials used in conventional medical devices. Additionally, the cost of 4D printers themselves remains a substantial barrier, as these machines are more complex and expensive compared to traditional 3D printers. As a result, the integration of 4D printing in healthcare faces the challenge of being cost-prohibitive for widespread adoption, especially in developing countries or for smaller healthcare providers with limited budgets.

**Figure 3:**
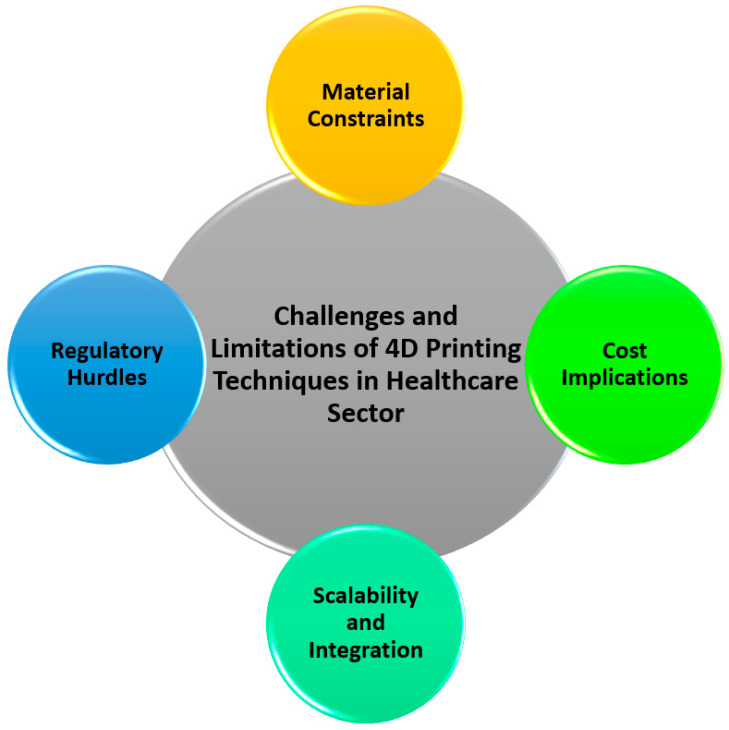
Challenges and limitations of 4D-printing techniques in healthcare sector.

Another significant hurdle is the scalability of 4D printing technology. While the production of custom, small-scale medical devices using 4D printing has shown promise, scaling up this technology for mass production remains a complex issue. Manufacturing large quantities of 4D-printed medical devices with consistent quality is challenging due to the variability in the behavior of responsive materials [59]. The adaptation of materials to specific stimuli such as temperature, moisture, or pH can lead to variations in their performance, which makes it difficult to ensure that each device meets strict regulatory standards for safety and efficacy. Furthermore, the complex, time-dependent transformation of 4D-printed materials adds another layer of difficulty in ensuring reproducibility during mass production [60]. To address these issues, it is necessary to develop advanced production techniques and improve the consistency of the materials used in 4D printing. A potential solution may involve a combination of automation, smart monitoring systems, and the optimization of the 4D printing process, enabling manufacturers to produce large volumes of reliable, cost-effective devices without compromising quality. Additionally, ongoing research into material innovations and cost-reduction strategies will play a crucial role in making 4D printing a viable solution for mass healthcare applications, ultimately driving its widespread adoption in medical practice. Figure 3 illustrates the aforementioned challenges.

### 4.4. Materials and Biocompatibility

Three-dimensional printing has immensely contributed towards facilitating the rapid prototyping and production of customized implants and prosthetic devices. However, 4D printing, apart from its numerous positive elements, presents some complexities that hinder its broad implementation. The primary limitation of 4D printing, in contrast to its predecessor, is the complexity involved in manufacturing dynamic medical devices that can react to external stimuli. In contrast to 3D-printed devices, which possess a static structure post-manufacturing, 4D-printed medical devices utilize smart materials that necessitate the meticulous regulation of their responsiveness to environmental variables, including temperature, moisture, or pH levels. This complexity presents some production challenges, as variations in material properties may result in inconsistencies in device performance. Scaling up laboratory-scale manufacturing to large-scale manufacturing of 4D-printed medical devices is not a simple process, largely due to challenges in achieving the consistency and replicability of stimuli-sensitive material properties. There is also a challenge in regulation that aggravates this limitation, in that current regulation largely deals with static medical devices, and there is a lack of frameworks to determine dynamically varying implants in their safety and efficacy over time. The cost represents a significant barrier to the widespread adoption of 4D printing in medicine, as the smart materials and advanced printing technologies necessary for their processing substantially elevate production costs relative to conventional 3D printing.

Future advancements in materials science are essential for enhancing the potential applications of 4D printing in the healthcare sector to address these challenges. A rising demand exists for advanced biomaterials that integrate high responsiveness with enduring stability and biocompatibility. Future research must prioritize the development of shape-memory polymers, hydrogels, and composite materials that exhibit improved durability and regulated degradation rates, thereby ensuring the reliable functionality of 4D-printed implants over prolonged durations. A crucial element of material innovation is the incorporation of bioactive compounds or drug-loaded nanomaterials into 4D-printed structures to facilitate tissue regeneration and tailored treatment approaches. Ensuring the biocompatibility of these materials presents a considerable challenge. Long-term studies examining the interactions of these materials with biological tissues, immune responses, and mechanical wear are crucial for regulatory approval and clinical adoption. Advancements in computational modeling and artificial intelligence may optimize 4D printing processes by predicting material behavior under physiological conditions, thereby enhancing precision and consistency. By addressing these concerns, 4D printing has the potential to exceed 3D printing in the development of smart, adaptive medical devices that enhance patient outcomes and reduce the necessity for surgical interventions.

## 5. Future Directions and Potential

As 4D printing technology continues to advance, its future potential in healthcare is significant, offering transformative possibilities for personalized medicine and surgical interventions. This technology, which enables materials to adapt and respond dynamically to external stimuli such as temperature, moisture, or pH, holds the promise of revolutionizing the development and functionality of medical devices. As the field progresses, new applications, materials, and processes are expected to emerge, further extending the capabilities of 4D printing in medical contexts. This section will examine the future directions and potential of 4D printing in healthcare, focusing on emerging trends, cutting-edge technological innovations, and the potential integration of artificial intelligence (AI) and machine learning (ML) to enhance the precision, customization, and efficacy of medical devices. The observed trends in the development of 4D printing for healthcare applications reveal a transformative shift in medical device innovation. Beyond the evident advancements in material adaptability and responsiveness, a deeper analysis highlights the pivotal role of interdisciplinary integration—where additive manufacturing converges with biomimetic materials, artificial intelligence, and machine learning. These synergies not only enhance the precision and personalization of medical devices but also redefine the way implants, prosthetics, and drug delivery systems interact with biological environments. Notably, the progression from conventional stents and implants to smart, self-adaptive medical solutions underscores a shift from static to dynamic therapeutic approaches. This evolution reflects both the promises and the persistent barriers of 4D printing in medicine, as material limitations, regulatory hurdles, and financial constraints continue to shape its trajectory. Understanding these complexities provides critical insight into the current landscape and sets the stage for the in-depth analysis presented in the following sections.

### 5.1. Emerging Trends

The future of 4D printing in healthcare holds immense promise, particularly in the fields of personalized medicine and surgical procedures. As the technology advances, one of the most interesting features is the development of customized medical devices and implants that can adapt to the unique needs of individual patients over time. This could include implants that change shape or properties in response to the patient’s specific biological conditions, such as stents that expand or contract based on vascular changes, or prosthetics that evolve in response to the patient’s growth or rehabilitation progress. In personalized medicine, 4D printing could be used to create highly specific, patient-tailored drug delivery systems that release medication in precise doses at targeted locations within the body, improving therapeutic outcomes while minimizing side effects. Furthermore, 4D-printed implants could be designed to gradually degrade or integrate with the body, minimizing the need for additional surgeries and reducing the risk of complications.

In surgical procedures, 4D printing could facilitate the creation of adaptive surgical instruments and tools that adjust during surgery to improve precision and reduce human error. For example, surgical guides could be 4D-printed to match a patient’s unique anatomy and then adjust during surgery to guide the surgeon in real time [61]. Additionally, 4D printing could enable the creation of dynamic scaffolds for tissue regeneration that respond to biological signals, promoting more effective healing. This adaptability could significantly improve the effectiveness of complex surgeries, particularly in areas like organ transplantation or reconstructive surgery. As the integration of 4D printing into healthcare continues to evolve, these emerging trends offer the potential to revolutionize how medical professionals approach treatment and care, providing highly personalized, adaptive solutions that improve patient outcomes and quality of life.

### 5.2. Technological Innovations

Ongoing research into new materials for 4D printing in healthcare is pushing the boundaries of what is possible in the creation of adaptive medical devices. A major area of innovation is the development of advanced biomaterials that can respond dynamically to various physiological stimuli. For example, researchers are focusing on designing materials that exhibit controlled shape-shifting properties, enabling them to adjust their structure in response to changes in temperature, pH, moisture, or even electrical signals. These materials are being explored for applications such as self-healing wound dressings, drug-delivery systems that respond to the body’s unique needs, and adaptive implants that adjust over time as they integrate with the surrounding tissue. Advances in hydrogels, bioactive polymers, and composites that combine biological materials with synthetic components are critical to advancing the functionality and biocompatibility of 4D-printed medical devices [62]. These innovations are laying the foundation for creating highly specialized, responsive devices that can more closely mimic the behavior of natural biological systems.

Alongside material development, new processes are being explored to enhance the precision, scalability, and efficiency of 4D printing technologies [63]. One promising area of research is the integration of multi-material 4D printing, which allows the creation of complex structures with a combination of materials that each react differently to stimuli [64]. This enables the fabrication of devices with more sophisticated functional capabilities, such as implants that can change shape at different rates in various areas of the body. Additionally, researchers are investigating more advanced printing techniques, including the use of light-based curing, bioprinting, and even microfabrication, to achieve finer resolution and more controlled responses from printed devices. The development of faster printing processes is also critical to making 4D printing a viable option for mass production, ensuring that customized medical devices can be produced at scale and at a reasonable cost. This evolution of the printing process plays a crucial role in bringing 4D printing closer to clinical applications.

Furthermore, researchers are working on integrating advanced systems that combine 4D printing with real-time data to enable adaptive medical devices that can actively respond to patient conditions [65]. For instance, incorporating sensors, wireless communication, and data analytics into 4D-printed devices could allow the continuous monitoring of a patient’s health and enable devices to autonomously adjust their functionality [66]. This could be particularly useful in areas like smart prosthetics or personalized implants that need to adapt as patients heal or undergo physical therapy. Artificial intelligence and machine learning algorithms are being employed to predict patient-specific responses and optimize the behavior of these devices in real time, further enhancing their therapeutic potential. As these technological innovations progress, they will not only make 4D printing in healthcare more efficient but also make the next generation of medical devices more intelligent, autonomous, and capable of delivering highly personalized treatments.

### 5.3. The Role of AI and Machine Learning

Artificial intelligence (AI) and machine learning (ML) will undoubtedly contribute to significantly enhance the precision and customization of 4D-printed medical devices, pushing the boundaries of personalized healthcare solutions [67,68]. These technologies can be integrated into the design and production stages of 4D printing to create devices that are not only more adaptive but also better tailored to individual patient needs. AI algorithms can process large volumes of patient-specific data, including anatomical and physiological information, to design 4D-printed devices with optimized functionality. For example, AI can assist in predicting how a 4D-printed implant or prosthetic will perform under varying conditions, based on real-time data such as temperature, pressure, or chemical environment. This capability is crucial for designing highly personalized implants that adjust over time, improving their fit and performance as patients heal or undergo rehabilitation. In essence, AI can act as a guide for the creation of devices that are not only anatomically correct but also functionally superior, adapting to the unique and dynamic needs of the patient.

In addition to aiding in the design process, machine learning models can contribute to the production of 4D-printed medical devices by improving the accuracy of the printing process itself [69]. ML algorithms can analyze the output from 3D printers and detect discrepancies or imperfections during printing, enabling real-time adjustments that ensure the highest level of precision. These algorithms can learn from previous print jobs and continuously improve the quality control process, reducing the likelihood of errors and enhancing the overall efficiency of the production pipeline. Furthermore, by integrating AI-driven predictive analytics into 4D printing, manufacturers could potentially develop systems capable of autonomously adjusting printing parameters based on the specific material, environmental conditions, or intended function of the device being printed. This adaptability ensures that the final product meets the desired specifications without the need for extensive human intervention, which is especially important in the highly regulated medical field.

Machine learning and AI also play a vital role in the ongoing use and monitoring of 4D-printed medical devices after they have been implanted. By incorporating sensors into the 4D-printed devices, real-time data on the device’s performance and the patient’s condition can be gathered and analyzed by AI systems. For instance, smart implants or adaptive prosthetics could continuously monitor variables like pressure, temperature, or movement, sending this data to healthcare providers or directly influencing the device’s behavior [70]. Machine learning models can then analyze these data to optimize the device’s function, learning from individual patient experiences and adjusting parameters over time. This integration of AI and ML into the lifecycle of 4D-printed devices represents a tendency towards fabricating truly autonomous, patient-specific medical devices that are capable of continuous improvement based on ongoing data collection, potentially leading to better clinical outcomes and reduced healthcare costs.

## 6. Conclusions

Four-dimensional printing has emerged as a groundbreaking technology with the potential to revolutionize healthcare by enabling the creation of dynamic, adaptive, and highly personalized medical devices. From self-healing implants that repair themselves over time to smart stents that respond to the body’s internal conditions, 4D printing has already shown significant promise in enhancing patient care and outcomes. The ability to design materials that react to stimuli such as temperature, moisture, or pH allows for the development of medical devices that can evolve alongside a patient’s healing process, offering unparalleled levels of customization and functionality. Personalized drug delivery systems that release medication in response to internal body conditions, as well as adaptive prosthetics and implants that improve over time, exemplify the diverse range of applications that 4D printing brings to healthcare. These advancements not only address the limitations of traditional medical devices but also open up new possibilities for more efficient, targeted, and effective treatments, leading to better patient experiences and outcomes.

However, while the potential of 4D printing in healthcare is vast, there are several challenges that must be overcome to fully realize its transformative potential. Material constraints, regulatory hurdles, and cost and scalability issues remain significant obstacles to the widespread adoption of 4D-printed medical devices. Biocompatible, responsive materials are essential for ensuring the safety and efficacy of these devices, and ongoing research is needed to develop materials that can meet these stringent requirements. Additionally, regulatory frameworks must evolve to accommodate the unique characteristics of 4D-printed devices, which may require new standards for approval and safety. The cost and scalability of 4D printing technology also pose challenges, particularly in terms of making these advanced devices affordable and accessible to a broad patient population. Despite these hurdles, the integration of AI and machine learning with 4D printing offers promising solutions to these challenges, allowing for greater precision, efficiency, and customization in the design and production of medical devices. As research and development in this field continue to advance, 4D printing is believed to become a key enabling fabrication technology of personalized, adaptive healthcare, assisting a multidisciplinary effort towards growing medical innovation.

## Figures and Tables

**Table 1:** Comparison of 3D and 4D printing in medical device evolution.

Aspect	3D Printing	4D Printing
Materials Used	Plastics, metals, ceramics	Hydrogels, shape-memory alloys, stimuli-responsive polymers
Applications	Prosthetics, orthopedic implants, surgical guides	Bio-implants, stents, drug delivery systems, adaptive devices
Key Features	Static components, customized fit, reduced time and cost	Dynamic components, stimuli-responsive, self-healing, adaptive to patient conditions
Benefits	Greater precision, lower manufacturing costs	Improved patient outcomes, reduced follow-up surgeries, real-time adaptation
Examples	Customized implants, surgical prototypes	Adaptive drug delivery systems, self-healing prosthetics, bio-implants integrating with tissues
Technological Impact	Enabled customization and rapid prototyping	Revolutionized healthcare with smart, responsive, and longer-lasting solutions
Future Potential	Enhanced precision in medical manufacturing	Next-generation personalized healthcare solutions, tissue regeneration, and advanced biosensors

**Table 2:** Comparison of the key applications of 4D printing in healthcare, including their benefits and current research examples.

Application	Description	Potential Benefits	Current Examples/ Research
Self-Healing Implants	4D-printed materials capable of repairing themselves when damaged, through a response to external stimuli (e.g., temperature, moisture).	- Reduced need for surgical intervention.	Self-Healing Implants
Smart Stents	Stents that respond to changes in body temperature or environmental factors, adapting to the body’s needs in real time.	- More effective treatment of blocked arteries.	Smart Stents
Personalized Drug Delivery Systems	Systems designed to release medication based on internal body conditions such as pH, temperature, or biochemical signals.	- Targeted drug delivery.	Personalized Drug Delivery Systems
Adaptive Prosthetics and Implants	Prosthetics and implants that can adapt and improve over time, responding to internal bodily changes or environmental conditions.	- Enhanced comfort and functionality.	Adaptive Prosthetics and Implants

## Abbreviations

The following abbreviations are used in this manuscript:

4D	Four-dimensional
3D	Three-dimensional
AI	Artificial Intelligence
FDA	Food and Drug Administration
CNC	Computer Numerical Control
ML	Machine Learning
CTE	Coefficient of Thermal Expansion
FDM	Fused Deposition Modeling
SLM	Selective Laser Melting
FDA	Food and Drug Administration
